# Soft Polymer-Based Technique for Cellular Force Sensing

**DOI:** 10.3390/polym13162672

**Published:** 2021-08-10

**Authors:** Zhuonan Yu, Kuo-Kang Liu

**Affiliations:** School of Engineering, University of Warwick, Coventry CV4 7AL, UK; z.yu@warwick.ac.uk

**Keywords:** soft polymer, hydrogel, force-sensing, cellular biomechanics, cell-friendly, 3D matrix, tissue engineering

## Abstract

Soft polymers have emerged as a vital type of material adopted in biomedical engineering to perform various biomechanical characterisations such as sensing cellular forces. Distinct advantages of these materials used in cellular force sensing include maintaining normal functions of cells, resembling in vivo mechanical characteristics, and adapting to the customised functionality demanded in individual applications. A wide range of techniques has been developed with various designs and fabrication processes for the desired soft polymeric structures, as well as measurement methodologies in sensing cellular forces. This review highlights the merits and demerits of these soft polymer-based techniques for measuring cellular contraction force with emphasis on their quantitativeness and cell-friendliness. Moreover, how the viscoelastic properties of soft polymers influence the force measurement is addressed. More importantly, the future trends and advancements of soft polymer-based techniques, such as new designs and fabrication processes for cellular force sensing, are also addressed in this review.

## 1. Introduction

Advancements in synthetic techniques have provided the possibilities for tailoring the structural compositions and the material properties of polymeric materials. Moreover, accompanied by the improvements in the imaging resolutions and the accuracy in the characterisations of mechanical properties, soft polymer-based materials have been increasingly favoured in the studies of tissue engineering and cellular mechanics [1,2]. Such materials are prevalent in the studies aimed for cellular force sensing, as their highly customisable mechanical properties and non-toxic nature can provide great versatility in such applications. In addition to cytotoxicity, as reviewed by Mondschein et al. [3], the consideration of factors, e.g., hydrolytic and enzymatic degradation properties, as well as the mechanical properties of soft polymer-based material, are needed for their adoption in biological studies. Various fabrication approaches for soft polymeric materials have been developed regarding biological applications, including moulding [4,5], casting [6], particulate leaching [7], electrospinning [8], gas foaming [9], and 3D printing [10,11]. Each of these fabrication processes has unique advantages and limitations in various aspects key to applying soft polymer-based materials in cellular force sensing, such as architecture, dimension, porosity, permeability and diffusion capabilities.

During cellular activities, such as growth, proliferation, migration, and apoptosis, cells not only sense and respond to biochemical signals, but also form mechanical interactions with their living environment [12]. These interactions between cells and the structures proximately surrounding them are expressed in the form of cell-generated mechanical forces, e.g., contraction forces. At the tissue level, cell-generated forces govern tissue developments through driving the repositioning of materials, as well as the bending and stretching of structures. Meanwhile, the forces also influence cellular processes, such as receptor signalling and transcription at the subcellular level [13]. As the properties of soft polymer-based materials can be tuned based on the needs, they have unique advantages in the application of cellular force sensing. Hence, the sensing of cellular forces through the adoption of soft polymers not only extends our understanding of the biophysical properties of living cells, but also supports the development of novel techniques and materials in the field of tissue engineering and biomedicine. Traditional techniques, such as atomic force microscope-based force spectroscopy [14,15], optical tweezers [16,17], magnetic tweezers [18], and force probes [19], have been extensively applied in measuring cellular forces. These state-of-art techniques offer great measurement accuracy. However, in comparison to the force-sensing technique utilising soft polymers, they have shortfalls in cell-friendliness and the ability to measure cellular force in a 3D matrix.

Soft polymer-based materials adopted in sensing cellular force usually take the form of hydrogels, where they can provide the cells with a physiologically relevant biomimetic 3D matrix. Hydrogels are water-swollen crosslinked polymeric networks that promotes cell function. The soft polymeric hydrogels can be customised versatilely in terms of stiffness, viscoelasticity, fluid content, oxygen permeability, and porosity. It can also be functionalised with specific bioactive molecules in accommodating and promoting particular cell types and cellular activities. In order for the soft polymer to be applicable in cellular force sensing, it is crucial to match its mechanical properties to that of native tissues. Within the human body, the stiffness of the extracellular matrix (ECM), where cells reside, can vary by several orders of magnitude between the softest brain tissue to the hardest bone tissue. However, cell types of interest in cellular force sensing are usually sourced from tissues with Young’s modulus ranging upwards of hundreds of kilopascal (10^5^ Pa), which falls within the elasticity range of soft polymeric materials. Thus, soft polymer-based materials shall serve as desirable materials for sensing cellular forces.

Over the year, both synthetic and natural soft polymers have been widely adopted for sensing cellular force. Based on synthetic material, techniques, such as deformable membrane (DM) [20,21], traction force microscopy (TFM) [22,23], and elastic-micropillar technique (EMP) [24], were developed. Meanwhile, naturally sourced polymers, such as collagen, agarose, and alginate, were also adopted with typical techniques of various collagen gel-based contraction assays (CGCA) [25,26,27,28] and culture force monitor (CFM) [29,30]. The working principles, merits and demerits of these techniques are summarised in Table 1. 

Recently, a number of reviews have been undertaken considering the available tools in sensing cellular forces [13,31,32]. These reviews provided detailed insights on the state-of-the-art techniques for measuring cellular forces, emphasising the accurate determination of forces generated by cells. In comparison, this review focuses mainly on how soft polymers can be utilised and optimised in sensing the forces generated by various types of cells by creating a cell-friendly environment in mimicking the biomechanical and physiological conditions of the tissue matrix. The advantages and limitations of each technique in regard to their quantitativeness and cell-friendliness will also be presented in the current review. Moreover, assessments of the viscoelastic properties of soft polymer-based materials are addressed and the prospects of the future application of these materials in the studies of cellular force sensing are provided.

## 2. Soft Polymer-Based Cellular Force Sensing Technique

Human cells generate and reside in a complex bioactive hydrogel scaffold (i.e., ECM). It comprises structural proteins such as collagen, fibronectin, and laminin, offering a mechanically stable micro-environment. Hydrated proteoglycan fills up the pores created by the structural proteins, acting as a storage and a medium for the diffusion of homeostasis-critical soluble molecules [33]. Cells exert and transmit internal tension and forces to the outside environment through cell-to-matrix adhesions. Such adhesions mainly occur at the sites of focal adhesions (FA), where the extended ends of cytoskeletal proteins aggregate join with FA proteins (e.g., talin, vinculin) through transmembrane receptors (i.e., integrins) [34]. Integrins will then bind to ligands present in the ECM, forming connections enabling the transmission of mechanical signals. Similarly, such connections can be formed between the cells and the soft polymeric material, where the force generated by the cells can be translated into the deformation of the substrate, constituting the basis of soft polymer-based techniques in cellular force sensing. In general, these techniques share a common underlying methodology. The characterisation of the mechanical properties of the soft polymeric substrate in conjunction with the assessment of the level of deformation of the substrate due to cellular force exertion will yield the amount of force generated.

### 2.1. Synthetic Soft Polymer-Based Techniques

With the increasing knowledge of synthetic polymers, many soft polymers have been applied to study cellular forces. The synthetic soft polymers in such applications share the advantages of being relatively easy in terms of mechanical property characterisation and substrate functionalisation in adapting for various cell types and application scenarios.

#### 2.1.1. Deformable Membrane (DM)

Harris et al. [20] developed a soft silicon rubber film-based technique to measure forces produced by cells. It was the first technique for assessing traction force produced by cells. When cells are seeded onto a thin silicon rubber film, the forces exerted by the cells cause visible wrinkles on the film. The length and patterns of the formed microscopic wrinkles are used to locate and follow the areas where cells are under contraction and extension. Moreover, the relative magnitude of force generated by different types of cells can be estimated [21]. Although it laid the foundation for measuring cellular force through the deformable substrate, this technique cannot quantitatively determine the magnitude and directions of the force. Further, the inhomogeneity of the thin film, such as surface defects and non-uniform thickness, can potentially introduce errors into the measurement [32].

#### 2.1.2. Traction Force Microscopy (TFM)

**Figure 1:**
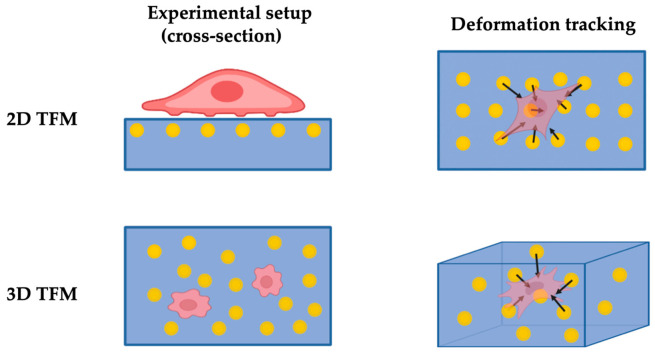
Schematics for TFM techniques (not to scale).

By better quantifying the polymer substrate deformation with the displacement of embedded microbeads, 2D TFM addressed the limitations of the DM technique. Pelham et al. [22] used a soft collagen-coated polyacrylamide hydrogel to study the locomotion of fluorescent-labelled cells seeded on the substrate surface. Based on this, Munevar et al. [23] improved on the quantitation and introduced the TFM technique by embedding a large number of fluorescent microbeads for measuring substrate deformation through visualising the bead displacements as shown in Figure 1. A typical TFM analysis involves optically imaging the bead distribution at certain time frames during force being exerted onto the polymer substrate. A computer algorithm maps the bead displacements and quantitatively resolves the force by incorporating the characterised substrate stiffness.

Over the years, TFM gained popularity as a versatile technique in sensing forces at the cell–matrix interface. The application of the technique spans many cell types such as osteoblasts [35], keratinocyte [36], smooth muscle cells [37,38], and HeLa cells [39]. As the sizes of the embedded fluorescent beads (≤1 μm) are typically much smaller than the scale of cells (tens of μm), it allows force distribution to be mapped with subcellular resolution [13]. The high resolution can serve to determine the contribution of active cytoskeletal contraction to traction force generation [40]. Moreover, the applications of TFM can be extended to measuring forces at focal adhesions [41] and traction forces during migration [23,42], as well as elucidate how pathological events influence the traction force [43,44]. Recently, stemming from the traditional 2D TFM technique, a number of techniques for visualising cell adhesive forces at greatly enhanced resolution have been developed. The visualisation is achieved through functionalising the soft polymer substrate surface by immobilising extracellular tension sensors, specifically targeting the focal adhesion transmembrane receptors [45,46]. The tension sensors are fluorescently labelled so that the variation of force is converted to the change of luminescence of the tension sensors, which results in the significant improvement of force resolution compared to the conventional TFM [47,48].

Despite the advancements, the 2D nature of the technique makes it far from ideal for promoting the physiological behaviours of cells. The in vivo environment for most cells is a 3D matrix instead of the surface of a 2D substrate. As a consequence of many cellular processes, cells behave very differently in terms of their mechanical characteristics when moved from an environment of a 3D matrix to the surface of a 2D substrate [49]. Thus, 3D TFM is developed to provide an additional dimension to address the limitations in 2D TFM, investigating traction fields and sensing cellular forces in more physiologically relevant conditions (i.e., cells embedded in a fibrous matrix). In essence, 3D TFM provides an additional dimension in the vertical direction over the 2D TFM through embedding cells and tracker beads within the matrix and measuring its deformation with embedded beads in all three dimensions.

The needs for tracking fiduciary markers in 3D constitute the majority of the added difficulties to 3D TFM, yet so far, several approaches have been developed. Bloom et al. [50] tracked the displacements of embedded fluorescent beads along the *z*-axis (vertical direction) using the patterns of out-of-focus diffraction rings in the optical system generated by beads. The tracking of the beads through this method can be accomplished through ordinary microscopy techniques. This approach requires only 5 s to track the bead displacements around the vicinity of a single cell, minimising measurement error by effectively ‘freezing’ the cellular motion during the measurement period. However, the spatial resolution along the *z*-axis is relatively low, at around 120 nm [50]. Laser-scanning confocal microscopy has become prevalent in the study of cell morphology due to its high-resolution imaging capacity in 3D, which also makes it a viable tool for tracking beads in 3D TFM. Legant et al. [51] first demonstrated 3D traction force measurement with polyethene glycol (PEG) hydrogel and confocal microscopy. It offers excellent spatial resolution, yet the acquisition process is slow. When optimised for resolution versus acquisition speed, the acquisition time for an individual cell’s traction field is approximately 3 min [51]. During the scan, changes to the matrix by cellular activities are beyond negligible, inevitably introducing uncertainty to the reliability of the results. Moreover, the reconstruction process of confocal microscopy-based 3D TFM is often very computationally intense [52]. Moreover, as observed by Maskarinec et al. [53], laser beams used in confocal microscopy are phototoxic to cells. Hence, a minimum of 30 min is required for acquisition interval, limiting its potential in dynamic cellular force sensing. Optical coherence microscopy (OCM), a variant of optical coherence tomography, can also be applied to track the movements of beads in 3D. The OCM offers rapid volumetric acquisition and utilises near-infrared wavelengths to mitigate light scattering and phototoxicity. However, such a system has a lower spatial resolution and is prone to introducing speckles [54].

Although many improvements in mimicking the in vivo environments have been made in the transition from 2D to 3D TFM, it still does not adequately represent physiological conditions. Firstly, cells are usually seeded at a very low density onto the polymer substrates to avoid interference of traction fields surrounding individual cells and minimise matrix remodelling [53,55]. As the generation of cellular forces is primarily regulated via mechanosensing, very low cell density will unavoidably hinder such processes. Secondly, unlike that in the 2D TFM, the soft polymer-based matrix serves both as a measuring device and a matrix to sustain cellular activities. In such a configuration, the polymer matrix will inevitably be subjected to local degradation, precludes the reconstruction of the matrix deformation and resolving the cellular force generation [50]. Thirdly, as the cellular force is determined through the measured elasticity of a homogenous, isotropic, and linearly elastic substrate, any errors in the process can be carried on and cause discrepancies in the resolved force. Lastly, the tracking of fluorescent markers is computationally demanding and often discrete [56,57]. This is especially true in recently developed super-resolved TFM techniques [58,59] as the density of the fluorescent bead has been dramatically increased to achieve 50–100 nm spatial resolutions compared to the 1–5 μm in the traditional TFM [60,61,62]. In the light of this, new techniques based on particle image velocimetry (PIV) [63] and particle tracking [64] have been developed to reduce computational costs in 3D TFM. Moreover, the solutions towards adding time dependency of marker tracking have been developed by Barrasa-Fano et al. [65] based on MatLab.

Overall, as a synthetic soft polymer-based technique, the limitations of TFM techniques primarily stem from its unsatisfactory cell-friendliness as a result of the type of polymer material used (e.g., polyacrylamide, PEG). While offering favoured properties such as being non-cytotoxic, isotropic, homogeneous, and time-invariant, there is a minimal resemblance of the ECM in biochemical aspects [13]. Attempts to use more physiologically relevant soft polymeric materials such as pericellular collagen have been made [50,55]. However, the nonlinear fibrillar nature of the collagen matrix prevents the application of classical mechanical approaches in resolving forces from local matrix deformations [13].

#### 2.1.3. Elastic Micro-Pillar Technique (EMP)

**Figure 2:**
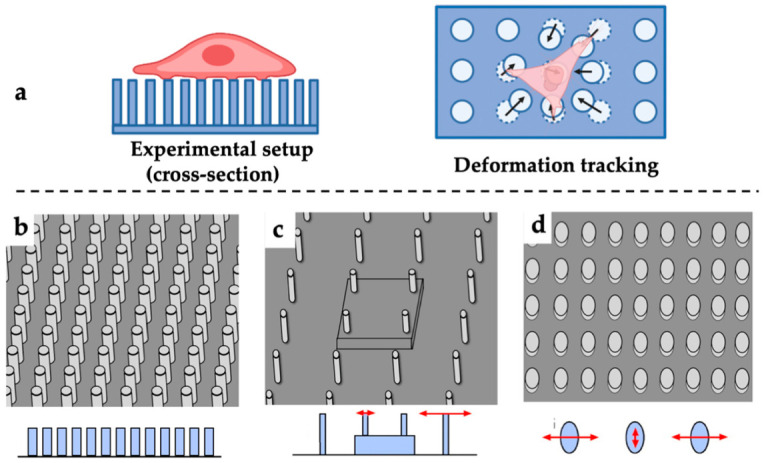
Schematics of EMP. (**a**) Methodology for cellular force sensing with EMP; (**b**) Tightly arranged short micro-pillars; (**c**) Loosely arranged micro-pillar matrix with varying heights; (**d**) Anisotropic pillar designs arrays.

In 2003, Tan et al. [24] developed microfabricated soft polydimethylsiloxane (PDMS) elastomeric arrays, i.e., microneedle-like posts, to spatially track the forces produced by cells attached on their tips (Figure 2a). Such a technique was invented as an alternative to TFM, as the beam deflections caused by cellular forces can be measured optically. The cellular forces can be determined by applying elastic beam theory as described by Equation (1), provided that Young’s modulus and dimensions of the micro-pillars are characterised.

(1) $$F=(\frac{3EI}{\;L^{3}})\delta$$

where *E*, *I*, *L*, and *δ* are Young’s modulus, the moment of inertia, length, and the horizontal deflection of the micro-pillar.

The EMP technique has seen widespread applications on many different types of cells, such as epithelial cells [66], endothelial cells [67], fibroblasts [68], various myocytes [69,70,71,72,73], and dendritic cells [74]. Compared with the TFM technique, this technique excels in the customisability of the micro-pillars while keeping the surface properties constant [24]. By controlling the length and diameter of the pillars, alteration of pillar compliance can be achieved. At the same time, the spacing between the micro-pillars can be adjusted for different cells types (Figure 2b,c). Meanwhile, variation in the moment of inertia of the pillars can be achieved for forces in different directions by changing the cross-sectional profiles of the micro-pillar (Figure 2d). Furthermore, various designs of the micro-pillar array will also affect how cells attach to, spread across, and deflect the micro-pillars. As the pillars deflect independently upon force exertion, the deflections of the micro-pillars directly reflect the subcellular distribution of traction forces [24]. Additionally, the micro-pillar arrays can be fabricated cost-effectively through casting [32].

However, such a technique also has disadvantages. Firstly, the synthetic soft polymeric nature and the array topology may stimulate undesired cellular responses. Secondly, as the micro-pillar deflection is optically assessed, the material choices of polymer substrate are limited to optically transparent materials, such as polydimethylsiloxane (PDMS) and polymethylmethacrylate (PMMA). Lastly, the casting process inevitably causes defects in pillar fabrication [75].

### 2.2. Natural Soft Polymer-Based Techniques

The utilisation of natural soft polymer for cellular force sensing has a unique advantage of best mimicking the physiological environment and promoting natural cellular behaviours. However, unlike synthetic soft polymeric materials, the characterisation of the mechanical properties of the natural soft polymer is not as straightforward and more prone to cellular remodelling. Moreover, compared to synthetic soft polymeric materials, natural soft polymers have limited potential to tailor the internal structure and alter mechanical properties.

#### 2.2.1. Collagen Gel-Based Contraction Assay (CGCA)

Within the body, cells are populated in the ECM, a 3D network of proteins (e.g., collagen, glycoproteins) and other biomolecules to provide structural and biochemical support to the cells [76,77]. Hence, the synthetic soft polymer-based cellular force sensing techniques lack cell-friendliness and may not truly reflect the in vivo cellular behaviours. As collagen is the most abundant matrix protein within the ECM [78], it undoubtedly becomes the prime candidate for designing hydrogel-based techniques for cellular force sensing.

In 1979, Bell et al. [79] introduced fibroblast-populated collagen lattice (FPCL) to study the contraction force generated by fibroblasts. They seeded fibroblasts into collagen hydrogel and measured cellular contraction force by observing gel shrinkage due to the force exerted onto the 3D cell-embedded collagen matrix. It provides excellent cell-friendliness over the synthetic polymer-based techniques in terms of sustaining three-dimensional physiological cellular interactions. Briefly, cells will form focal adhesions and exert traction force onto collagen fibrils during the spread within the collagen matrix. Since the collagen fibrils within the matrix are linked and intertwined, forces exerted onto individual fibrils will propagate and cumulate to cause global contraction of the collagen matrix. To construct the contraction assay, cells are seeded into the collagen solution with desired cell density and collagen concentration at 4 °C. The seeding is followed by a polymerisation period of approximately 20 min, after which culture media is added to provide nutrition to the embedded cells. The gel is often subsequently dislodged from the bottom of the Petri-dish and allowed to contract without constraints while suspended in media. The protocols regarding the construction of the assay are documented in detail by Ngo et al. [80]. 

**Figure 3:**
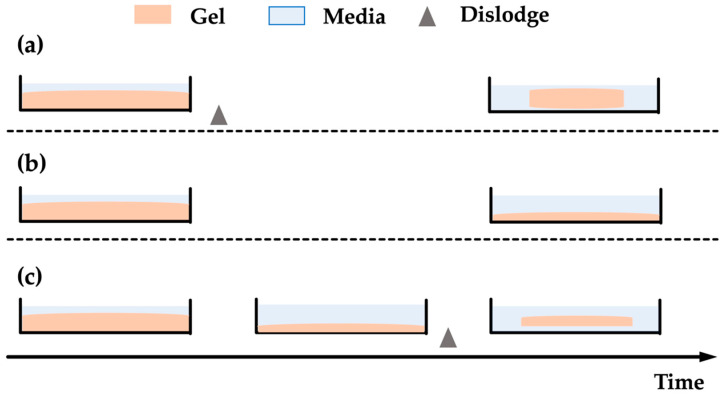
Schematics showing the variation of contraction assays based on dislodge time. (**a**) Immediate dislodge; (**b**) No dislodge; (**c**) Dislodge after a period of time.

Cell contraction is detected by measuring the percentage reduction of gel area after a period of culturing with optical systems [79]. Since the first introduction, several variations to the technique have been developed with varying timing of dislodgement (shown in Figure 3). The most common method is to dislodge the cell-embedded gels after polymerisation immediately (Figure 3a) and obtain measurements after a period of contraction. Such a method will result in the vast majority of the gel shrinkage being along the radial direction. Figure 3b demonstrates a variation of the methodology where no gel dislodge occurs. As the gel remains in contact with the rigid Petri-dish, the resultant tension within the gel will primarily reduce the gel thickness. In comparison, the former method provides a more straightforward measurement with an optical system than the latter. Figure 3c shows a combined method, where a period of culturing is allowed to elapse before dislodging. Such a method was invented and widely used to study how external stress affects cellular force generation, as internal stress accumulates due to the force exerted on the polymeric gel matrix prior to the dislodgement [81,82].

In the early 1990s, Moon et al. [26] developed a novel approach known as fibroblast-populated collagen microspheres (FPCM) to sense cellular force by probing the interactions between cells and collagen fibrils. In comparison, the FPCM is a spherical analogue of the traditional FPCL proposed by Bell et al. Instead of a disk-shaped hydrogel, a gel sphere is constructed by pipetting cell-containing gel solution into a silicone fluid at 37 °C. The novel approach provides several advantages over the traditional approach. Firstly, the spherical design offers a more straightforward way of assessing the forces exerted on the collagen gel. The measurement will be one-dimensional (i.e., axisymmetrical) instead of two-dimensional as required for the disk-shaped gel. The spherical geometry also enables the contraction of FPCM to be mathematically simplified, enabling the model to be described with only a spherically symmetric set of equations. Secondly, as Modis et al. [83] reported, the methodology applied in constructing traditional disk-shaped gel can lead to a significant local anisotropy in the collagen matrix. This is recognised to be a result of fibrillogenesis in the presence of bounding surfaces. Whereas the collagen microsphere in the FPCM technique is prepared by pipetting the gel solution into silicone fluid, the initial collagen fibril orientation is relatively isotropic. Lastly, the diameter of the yielded soft polymeric microspheres can be arbitrarily small with a typical value of 1 mm [26], allowing a minimised diffusion gradient for mass transport. 

Due to the relative ease of fabricating and the cell-friendliness resulting from its natural soft polymer-based material, many studies have adopted CGCA for sensing cellular forces of cells of different physiological origins. The technique has seen extensive usages among the study of cellular forces in pathological states, such as cardiovascular disease [84], respiratory disease [85,86], eye disease [87], as well as cells during physiological events, such as ageing [88], and wound healing [89,90,91].

**Figure 4:**
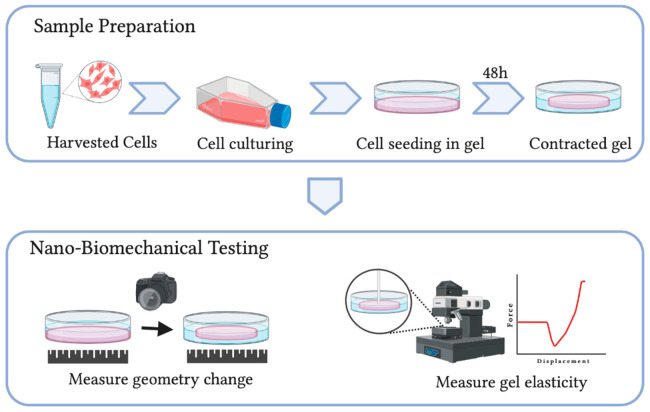
Schematic shows the methodology of the new nano-biomechanical technique for cellular force measurement.

Despite its cell-friendly merit and the ability to simulate various physiological conditions, conventional CGCA techniques can only provide a qualitative assessment of cellular forces. The mechanical properties of the soft polymeric matrix, such as the elasticity of the collagen matrix, are unknown. Recently Jin et al. [27] developed a new nano-biomechanical technique on cell-embedded collagen hydrogels in combination with mathematical modelling, which measures both elasticity and geometrical changes of the polymeric gel in determining cellular forces (Figure 4). The new technique provides merits in quantitativeness without sacrificing cell-friendliness. With such a technique, the influences of matrix stiffness [92], and ageing [28] on cellular force generation were quantitatively studied.

Considering that cells constantly remodel their surrounding matrix, the capability of characterising matrix stiffness carries even greater importance in the natural polymer-based force sensing techniques. This is especially true for certain cell types, such as fibroblasts and chondrocytes, as their functions are primarily dependent on matrix remodelling and the dependency becomes pronounced during states such as ageing [93,94].

#### 2.2.2. Culture Force Monitor (CFM)

As reviewed above, conventional CGCA techniques offer a cell-friendly and straightforward approach to assess cellular forces with 3D soft polymeric hydrogels. Apart from its non-quantitative feature, it also lacks measuring sensitivity as it cannot effectively display observable geometric change under small force output. To improve on the technique, Delvoye et al. [29] developed a CFM system to achieve direct measurements of cellular forces by attaching a force transducer on the edge of the cell-embedded collagen gel.

**Figure 5:**
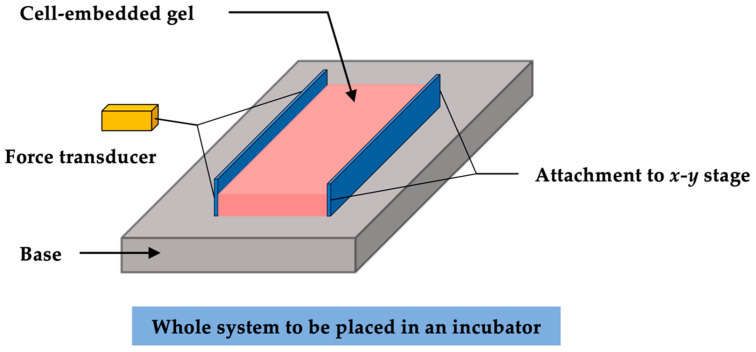
Schematic diagram of a typical CFM system.

In a typical CFM system, the sample gel is fixed to two diametrically opposed plates connected to a force transducer and translational stage, respectively. The stage is used to pre-stretch the gel prior to the start of the measurement (Figure 5). The entire system is placed within a standard culturing incubator and a wireless transducer acquires the force measurements. Alternatively, needle-like force transducer probes can be attached isometrically on the free-floating collagen hydrogel and differential measurements can be taken between transducer pairs. Sensitivities up to 0.5 mm and 0.5 mN can be achieved on the displacement and force, respectively [30,95]. The most prominent benefit of the CFM technique is its ability to allow precise, high sensitivity measurements of multi-cellular force directly in a physiologically relevant environment (i.e., in hydrogel matrix) and continuously monitor the force changes throughout the culturing period. Moreover, the system enables the easy application of external mechanical stimuli, providing the ability for more complex experimental design [32]. Recently, significant progress has been made by Campbell et al. [30] and Peperzak et al. [96] to increase the efficiency of CFM. The multi-station dynamic CFM allows multiple gel samples to be measured simultaneously, significantly reducing the measuring time and providing better variable controls throughout the study. Many studies have adopted CFM to measure different cell types, such as fibroblasts [97,98], endothelial cells [98], and cardiac myocytes [99].

However, there are still several disadvantages associated with CFM. One of the main disadvantages is system complexity. The setup will inevitably disturb the normal cellular processes, adding uncertainties to the result and increasing infection in the measuring process. Moreover, axially constrained cellular force measurements, such as CFM experiments, often have a limited measurement time due to the eventual mechanical homeostasis [100]. Additionally, as the force is acquired from a discrete amount of force transducers, the interpretation of the result is dependent mainly on the transducer placement. Lastly, the setup for such a system is relatively complicated.

## 3. Dynamic Interactions between Cells and Soft Polymer-Based Matrix

Cells are continuously remodelling their surrounding matrix, altering the surrounding mechanical and chemical environment. These changes reciprocally regulate cellular processes through mechanosensing. Such a process provides the basis of matrix elasticity influencing cellular behaviours. It has been noticed that cell’s interactions with the ECM can result in the display of a different phenotype in vivo compared to that defined otherwise by their genotype [101]. Studies on glioma cells show that the interactions with a stiffer polymer matrix can promote cell division, where the division rate increased by five-fold compared with a softer matrix [102]. Moreover, the sudden changes in the stiffness of the soft polymer-based matrix are an important factor in promoting malignant transformation, tumorigenesis, metastasis [103]. The stiffness changes of matrix are also related to the changes of cytoskeletal tension in tumour cells [104]. Moreover, the behaviours of specific cells on a soft polymer substrate with altered stiffness is characteristic of particular phenotypes. For example, cancerous cells can be distinguished by their ability to grow on substrates softer than their corresponding healthy tissues [105]. Such a discovery suggests that a cell does not act as a solitary individual, and any cellular behaviour should be assessed in the context of the surrounding cells and their matrix. It also presented a promising perspective in applying soft polymer-based materials in the study of cellular behaviours as the dynamic matrix will trigger intercellular signal transduction in the regulation of cell behaviours.

The stiffness of a homogeneous soft polymeric material is dependent on the concentration of the polymer and the crosslinking density. The influences of polymer matrix stiffness can vary between different cell types. Marklein and Burdick [106] demonstrated increased proliferation and migration of mesenchymal stem cells (MSC) on 3D hyaluronic acid scaffolds with higher stiffness, while Banerjee et al. [107] concluded the opposite for neuronal stem cells. In terms of the cellular force measured using a soft polymer-based matrix, fibroblasts in stiffer collagen hydrogel have shown reduced contraction [92]. However, the influences of polymer matrix stiffness in cellular force sensing cannot be simply assessed in isolation. As a dynamic system, cells embedded in the soft polymeric matrix constantly remodel their surroundings, changing the matrix stiffness. The remodelling rate depends on the initial stiffness of the matrix, demonstrated separately by Zhu et al. [108] and Ahearne et al. [109] on fibroblasts with lung and corneal origins. Based on the mechanism of cell-mediated matrix contraction, if the polymer fibrils buckle under the force applied by the cells, they cannot reciprocally stimulate cellular force generation. The lack of stimulation will result in decreased cellular force output. It suggests that polymer matrix with low stiffness tends to reduce the cellular force generation as it is more likely for the polymer fibrils to buckle under increased traction force due to reduced fibril density. Moreover, the difference in matrix stiffness can affect properties key to cellular activities such as the number of focal adhesion sites, oxygen and nutrient permeability. Such a complication also indicates the benefits of adopting soft polymer-based techniques in cellular force sensing, where physiological cellular behaviours are preserved.

## 4. Viscoelastic Properties of the Soft Polymer Matrix

Viscoelasticity is essential for biological functioning as it plays an important role in the storage, transmission, and dissipation of forces and energy within living tissues [110,111]. Soft polymer-based techniques in sensing cellular force often require the construction of polymeric hydrogels. Polymeric hydrogels often contain a large amount of water, thus exhibit viscoelastic behaviours. Moreover, based on the molecular structures of their polymer chains, the viscoelastic properties vary. Collagen hydrogels are shown to be significantly viscoelastic among few commonly used natural hydrogels (e.g., agarose and alginate). How the viscoelasticity of the hydrogel affects the embedded cells remains unclear [112], yet it has been shown to promote cellular behaviours not seen on purely elastic matrix [113]. The effects of viscoelasticity on embedded cells have also been shown to change alongside cellular force exertion and matrix remodelling [109]. The application of soft polymer-based material in cellular force sensing requires accurate characterisation of the mechanical properties of the polymer matrix. As a soft polymer, often, the viscoelastic property manifests in its force-displacement response when applying such techniques. Therefore, characterisation of the viscoelastic properties of soft polymer-based material is essential when applying for sensing cellular forces.

Viscoelastic materials are materials that display both elastic and viscous characteristics simultaneously at a considerable level when undergoing deformation [114]. Similar to soft biological tissues, polymeric hydrogels are typical biphasic materials where a solid network forms the scaffold of the structure with water filling up the porous cavities [115]. It is commonly believed that the solid polymer network is responsible for the elastic characteristics of the hydrogel, whereas the network mobility and fluid flowing within the network contributes to the viscous properties of a viscoelastic material [116]. Within polymeric hydrogels, the interstitial fluid phase (e.g., water) can be categorised into two groups: ‘free-flowing’ and ‘fixed’. The ‘free-flowing’ water can easily undergo material exchanges and diffuse in and out of the hydrogel. The ‘trapped’ water (i.e., fixed phase) is tightly bound to hydrophilic fibrils through hydrogen bonding. It has an important role in stabilising the polymer matrix structure within the hydrogel and contributing greatly to the viscous properties of the hydrogel. At the same time, the hierarchical structure of polymer fibres provides the elastic strength of the hydrogel.

The major approaches for characterising viscoelastic behaviours are through measuring stress relaxation and creep responses. The stress relaxation test applies a fixed deformation to the sample and measures the stress-time response of the sample, while the creep test applies a fixed force and records the strain-time curve. The time-dependent stress and strain data from the tests is subsequently used to derive viscoelastic parameters from constitutive models. Tensile stress relaxation tests are among the most popular in determining the viscoelastic responses of materials, yet the nature of biomaterials, especially hydrogels, does not necessarily permit such tests to be performed.

In the last decade, many alternative mechanical testing approaches have been developed to suit the specific needs in characterising hydrogels materials. Ahearne et al. [117] demonstrated the mechanical characterisation of viscoelastic properties of biomimetic membranes through micro-shaft poking. They also proposed a novel method of dropping a stainless steel ball onto a thin, soft polymeric hydrogel film [118]. Cheng et al. [119] and Mattice et al. [120] demonstrated the technique for stress relaxation and creep tests with spherical indentation, allowing samples to be placed in an aqueous medium. Although, compared with other methods, the indentation methods are confined to measuring localised viscoelastic response rather than the entirety of the material, it offers the benefit of causing minimal disturbance to the soft polymeric hydrogel.

**Figure 6:**
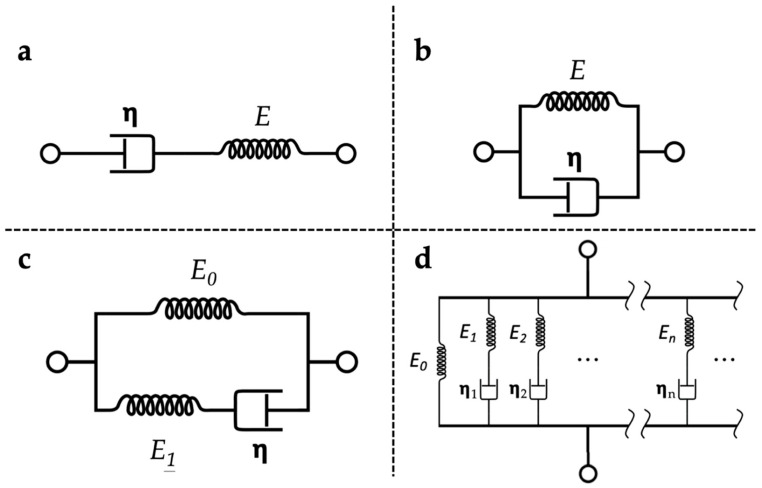
Schematics of typical constitutive models for viscoelastic materials. (**a**) Maxwell model; (**b**) Kelvin-Voigt model; (**c**) Standard linear solid (SLS) model; (**d**) Maxwell-Wiechet model. *E* and *η* are the elastic modulus of the elastic spring element and the viscosity of the damper element respectively.

Many constitutive models were developed for describing the viscoelastic behaviour of materials. It has been well recognised that the Maxwell model [121] (Figure 6a) and Kelvin–Voigt model [122] (Figure 6b) offer simplicity cannot yet achieve satisfactory predictions on the stress relaxation behaviours on soft polymeric materials [123]. The standard linear solid (SLS) model [124,125] (Figure 6c) addresses the limitations of both Maxwell and Kelvin–Voigt models by combining the elements of the two. The application of the SLS model is suitable for both creep and stress relaxation analysis, which made it one of the most common viscoelastic models in studying highly hydrated soft polymer-based materials [115]. Moreover, a more general form of the linear viscoelastic model [126] (Figure 6d) is available by the addition of ‘Maxwell elements’ to the model. The addition enables the distribution of relaxation times in the model to achieve more realistic relaxation modelling. A study has shown that a generalised SLS model (i.e., Maxwell–Weichert model) can provide a more accurate representation of the relaxation response than the SLS model [117]. However, as extra calculations are required with more unknown parameters, the added complexity outweighs the gain of accuracy in characterising the viscoelastic properties of soft polymeric hydrogels.

The viscoelastic behaviours of soft polymer-based materials post additional complexities in characterising their mechanical properties to sense cellular force. This not only requires additional considerations towards the process of mechanical characterisation, but also demands a nonlinear mathematical model for the technique. A numerical simulation study conducted by Yu et al. [28] based on the finite element methods on the soft collagen gel suggests that with less than 10% strain in the deformation, the polymeric collagen hydrogel can be treated as a linear-elastic material. The methodology and findings provide a feasible route for tackling viscoelastic behaviours presented by soft polymer-based materials during the application in cellular force sensing.

## 5. Intricate Problems in Cellular Force Sensing

### 5.1. 2D Substrate or 3D Matrix

A common approach for in vitro study of cellular behaviours is to adopt a 3D cell-embedded polymeric hydrogels matrix. A difference can be found between the traditional 2D approach and the 3D hydrogel matrix in how cells sense the micro-environment. For instance, cells are only partially in contact with the substrate and the neighbouring cells on a 2D substrate, causing polarised mechano-transduction and unnatural behaviour of the cells [127]. The rest of the cell surface that is not in contact with the substrate and neighbouring cells directly contact the culturing media. Despite direct nutritional and waste exchange with the media, the homogeneous culture media does not create a concentration gradient of nutrients, growth factors, and cytokines as observed for in vivo conditions. It is shown that the dynamic spatial concentration gradients of soluble factors in the ECM have influences on cell migration, communication, and differentiation.

Thus, for the sensing of cellular forces, a 3D soft polymer-based culturing model should be used to best recapitulate the mechanical and biochemical stimulation present in native ECM. As natural soft polymers offer great cell-friendliness, the 3D scaffolds constructed with such soft polymeric materials can best mimic native ECM, promoting physiological behaviours of the cells. The benefits of using a 3D hydrogel-based technique in sensing and differentiating cellular forces in different physio-pathological states (e.g., ageing fibroblasts, chondrocytes) are especially prominent, as the ageing of fibroblasts is a complex process involving changes in many aspects which change its matrix remodelling and mechanosensing abilities [128].

### 5.2. Measurement Accuracy

Usually, quantitative analysis involves measuring the displacement response of an applied force to the soft polymer-based substrate, thus requiring measurement instruments with resolutions in the scale of milli-newton (mN) and micro-meter (μm) for force and displacement, respectively. At such a fine resolution, the instruments are inevitably prone to environmental perturbations. Moreover, in macro-scale mechanics, it can be reasonably assumed that materials are homogeneous and that the size and shape of the probe are negligible. In the cellular force sensing techniques that utilise a soft polymer-based matrix, the heterogeneity in the matrix caused by the cell remodelling and force exertion, as well as the geometrical profiles of the probe, can no longer be ignored. However, both issues can be addressed by applying a relatively simple mechanical model and making an appropriate assumption based on the aim of the study.

## 6. Prospects

Soft polymer-based materials have been widely used in sensing cellular forces to promote natural cellular behaviour by providing cells with physiological environments. As the fabrication approach and mechanical characterisation of soft polymer-based materials undergo further developments, the utilisation of soft polymer-based materials broadens. A few trends regarding future applications which can be envisaged are reported here.

### 6.1. Extended Choice of Natural Materials

Natural soft polymer material has the unique advantage of mimicking the physiological environment and promoting natural cellular behaviours. Conventionally, due to the obtainability and easiness of handling, the choices of natural polymers for cellular force sensing are limited to polymers such as collagen, agarose, and alginate. Recently, more naturally sourced soft polymers, such as fibrin [129,130,131], hyaluronic acid (HA) [132,133,134], and fibronectin [135], have been adopted in the construction of 3D soft scaffolds for tissue engineering. With the extended range of naturally sourced polymer materials, the potential of using soft polymer-based techniques in cellular sensing can be increased.

### 6.2. Bioactive Modification of Synthetic Materials

Soft polymeric hydrogel is an attractive material for mimicking natural ECM. However, the methodology can be further improved with bioactive modification to better address the biochemical composition of various tissue at different physiological and pathological states. Zhu et al. [136] reviewed that short peptide chains derived from ECM proteins such as laminin and fibronectin, are among the popular choices for bioactive modification of PEG hydrogels. Petrini et al. [137] have also reported the design and functionalisation of polyurethane hydrogels for tissue engineering. A broad range of potential studies can be conducted with the bioactive modification of the soft polymeric matrix. For example, the cell-embedded hydrogel can be bioactively modified to change the state of crosslinking based on the morphological or the physiological state of the embedded cells. The change of crosslinking structure of the hydrogel will intrinsically alter the mechanical properties of the hydrogel, which may trigger specific force response of the cells, adding diversity to the force sensing techniques based on soft polymer-based materials.

### 6.3. 3D Printing of the Soft Polymer Scaffold

3D bioprinting techniques have emerged in the past decades, capable of producing tailored structures within the biomimetic, complex, and cellularised 3D soft polymer scaffold appropriate for desired cell populations [138]. As reviewed by Li et al. [139], 3D printed soft polymer network can facilitate matrix remodelling, migration, and adhesion, which are desirable in cellular force sensing applications. Compared with the current soft polymeric assays, the 3D printed tissue-equivalent will be a significant leap in advancing the technique. It will ultimately represent the conditions of native tissues and promote cellular behaviours closest to in vivo conditions. Moreover, the customisability of 3D bioprinting has the potential for replicating pathological tissues, which could be the next advancement in polymeric material-based cellular force sensing

### 6.4. Improvements of Methodological Design on Soft Polymers

**Figure 7:**
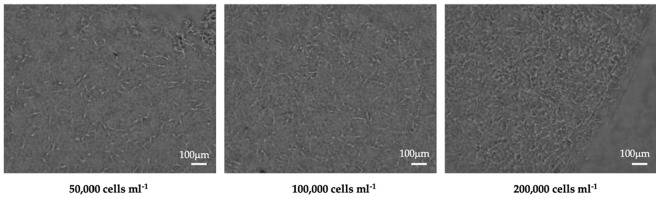
Brightfield microscopy images of human dermal fibroblasts in collagen hydrogel matrix with three different seeding densities. Images showing cell morphology post 48h incubation period.

Several methodological approaches can be taken to improve the performance of soft polymers in force sensing applications. Firstly, the polymer matrix density and cell seeding density can be optimised to form a cell-friendly biomimetic 3D matrix. As shown in Figure 7, at both seeding densities of 50,000 cell ml^−1^ (low) and 100,000 cell ml^−1^ (medium), cells had enough space to spread and separate from each other, while at 200,000 cell ml^−1^ (high), they are generally aggregated and attached with neighbouring cells. A 26% reduction in contraction force generation was observed on samples with high density due to the saturation of adhesion sites on the collagen matrix [28]. The results show that the polymer matrix density and cell seeding density adopted in the techniques need to be extensively designed to provide cells with environments closest to in vivo conditions, improving their performance in cellular force sensing.

Secondly, as reviewed above, the viscoelasticity of the cellular matrix regulates fundamental cellular processes and promoting cellular behaviours otherwise not seen in both 2D and 3D cultures [113]. This offers a perspective in tuning the viscoelasticity of the soft polymer matrix to improve the force-sensing capability. The viscoelasticity of a polymer is primarily determined by the structure and level of crosslinking of the polymer network, as well as the type of crosslink bonds. In an ideal covalently crosslinked polymer network, the energy will only dissipate through the uncrosslinked loose ends of the polymer, while the non-ideally crosslinked polymers will lead to creeping [140,141]. Altering the ratio between monomer and crosslinker is a viable approach to change the crosslinking structure. As Charrier et al. [142] demonstrated on polyacrylamide hydrogels, the variation of storage and loss moduli can be tuned by this approach. Moreover, the strength of the crosslinking bond can also determine the viscoelastic properties of the soft polymer matrix as shown on PEG [143,144], alginate [145], and peptide-based hydrogels [146]. Crosslinking bond strength can be controlled through modifications to polymer chains, such as changing molecular weight, inserting inert molecules to control polymer section length and changing the affinity of weak bonds (e.g., hydrazine, boronate bond). By modifying polymer structures, the viscoelastic properties of the soft polymer can be customised to suit the needs of cellular force sensing better.

Lastly, soft polymers can be fabricated in conjunction with bioelectronics to convert the matrix deformation to electric signals. As reviewed by Boys and Owens [31], bioelectronics has great potential in cellular force sensing. Several innovative designs for the EMP technique have seen the usage of magnetised pillars in exerting forces on culturing cells [147,148], which propose a feasible methodology for using conducting soft polymers to sense substrate deformation in the form of capacitance or impedance changes.

## 7. Conclusions

This review described the importance of force generation in cellular activities and emphasised the necessity and importance of accurately determining cellular forces. We highlighted the advantages of utilising soft polymer-based materials based on their benefits in preserving dynamic cell–matrix interactions. We outlined the influences of the viscoelastic behaviours of the soft polymeric matrix on embedded cells and provided common approaches to characterising such behaviours. Lastly, we presented prospects on the future trends and advancements of polymer-based cellular force techniques, as well as several methodological designs for improving the performance of the soft polymers for cellular force sensing.

## Figures and Tables

**Table 1:** Comparison of common soft polymer-based cellular force sensing techniques. (DM: deformable membrane; TFM: traction force microscopy; EMP: elastic-micropillar technique; CGCA: collagen gel-based contraction assays; CFM: culture force monitor; PEG: polyethene glycol; PDMS: polydimethylsiloxane; PMMA: polymethylmethacrylate).

Polymer Origin		Polymer Type	Principles	Advantages/ Disadvantages
Synthetic	DM	Silicon rubber	Length and patterns of wrinkles on film shows force generation	Simple and cheap/ Not cell-friendly; not quantitative
	2D TFM	Poly- acrylamide; PEG	Use fluorescent microbeads to track substrate deformation due to cells seeded on the surface	Highly quantitative/ Not 3D; not cell-friendly
	3D TFM		Tracking of matrix deformation due to embedded cells in 3D	Highly quantitative, 3D/ Not mimicking in vivo environment; computationally extensive
	EMP	PDMS; PMMA	Optically measure the deflection of micro-pillar array	Highly quantitative/ Not 3D; not cell-friendly
Natural	CGCA	Collagen gel	Measure the geometry of cell-embedded collagen hydrogel	Highly cell friendly; 3D/ Qualitative
	CFM	Collagen gel	Continuously measure the force generated through attached strain gauges	3D; qualitative/ Complex; disturbance to cells

## Data Availability

The data presented in this study are available on request from the corresponding author.
